# Cardiac Arrest as an Uncommon Manifestation of Late Type A Aortic Dissection Associated with Transcatheter Aortic Valve Replacement

**DOI:** 10.3390/jcm12165318

**Published:** 2023-08-16

**Authors:** Jan Naar, Dagmar Vondrakova, Andreas Kruger, Marek Janotka, Iva Zemanova, Martin Syrucek, Petr Neuzil, Petr Ostadal

**Affiliations:** 1 Department of Cardiology, Na Homolce Hospital, 150 30 Prague, Czech Republic; dagmar.vondrakova@homolka.cz (D.V.); andreas.kruger@homolka.cz (A.K.); marek.janotka@homolka.cz (M.J.); pneuzil@seznam.cz (P.N.); petr.ostadal@homolka.cz (P.O.); 2 Department of Pathology, Na Homolce Hospital, 150 30 Prague, Czech Republic; iva.zemanova@homolka.cz (I.Z.); martin.syrucek@homolka.cz (M.S.)

**Keywords:** aortic dissection, cardiac arrest, extracorporeal cardiopulmonary resuscitation, transcatheter aortic valve replacement

## Abstract

Transcatheter aortic valve replacement (TAVR) is a minimally invasive therapeutic procedure with a consistent, linear increase in the number of implantations worldwide. Recently, TAVR has been rapidly expanding into lower-risk populations. Sporadic cases of late prosthesis-related Stanford type A dissection have been documented in self-expanding, as well as balloon-expandable TAVR valves, manifested primarily as acute aortic syndrome. We present the case of a 76-year-old male, who experienced refractory in-hospital cardiac arrest with non-shockable rhythm due to the obstruction of coronary flow caused by aortic dissection type A, with entry directly adjacent to the aortic prosthesis according to autopsy. The patient died despite the engagement of extracorporeal cardiopulmonary resuscitation. Aortic dissection developed one year after a transfemoral TAVR procedure using an Edwards SAPIEN 3 29 mm self-expanding valve. TAVR-associated late aortic dissection type A represents a rare, life-threatening condition with various clinical manifestations. The risk factors have not been well described and the differential diagnosis may be challenging. As the number of TAVR recipients and their life expectancy is increasing, we may face this complication more often in future.

## 1. Introduction

Transcatheter aortic valve replacement (TAVR) is an evolving, minimally invasive procedure indicated in severe degenerative aortic stenosis, with a consistent increase in the number of implantations in developed countries [1]. Thoracic aortic dissection (TAD) represents an infrequent periprocedural complication [2]. Rare cases of late TAD Stanford type A following the TAVR procedure with dissection entry localized in the area of the prosthesis have been reported [3,4]. Late TAVR-associated TAD typically presents as acute aortic syndrome with chest and/or back pain as leading manifestations. Nevertheless, late TAD may be an underdiagnosed cause of cardiac arrest in TAVR recipients. Rarely, TAD may also occur as a consequence of cardiopulmonary resuscitation-induced trauma [5]. In some cases of sudden death, it can be difficult to determine, whether TAD represents an underlying condition that led to cardiac arrest, or was a secondary, traumatic, cardiopulmonary resuscitation-induced condition [6]. We report a case of refractory in-hospital cardiac arrest treated by extracorporeal cardiopulmonary resuscitation, where the cause of circulatory collapse was revealed with substantial time delay and the causal mechanism was definitively identified on autopsy.

## 2. Detailed Case Description

A 76-year-old Caucasian male, body mass index 39 kg/m^2^, with a history of an uncomplicated transfemoral TAVR procedure for combined degenerative aortic valve disease (severe stenosis, mild regurgitation) 12 months prior was admitted to the ophthalmology department for cataract surgery. The Edwards SAPIEN 3 29 mm valve (Edwards Lifescience, Irvine, CA, USA) was used. The patient was former cigarette smoker, with a history of arterial hypertension, diabetes mellitus type 2, permanent atrial fibrillation, chronic obstructive pulmonary disease with mild airflow obstruction, who experienced severe bilateral community-acquired pneumonia of unknown origin 5 months prior, which required 7 days of mechanical ventilation.

The previous TAVR procedure was followed by improvement in clinical manifestations (reduction of exertional dyspnea). Examination by a cardiologist, that preceded cataract surgery by three days, documented favorable clinical status, persistent atrial fibrillation, heart rate roughly 70 bpm, and systemic arterial pressure 115/70 mmHg. According to transthoracic echocardiography, there were no signs of TAVR prosthesis malfunction (systolic pressure gradient 16/10 mmHg, mild regurgitation). The left ventricle was non-dilated, with mild concentric hypertrophy and an ejection fraction of 50%. Aortic root diameter was 41 mm, and ascending aorta diameter 46 mm.

**Figure 1:**
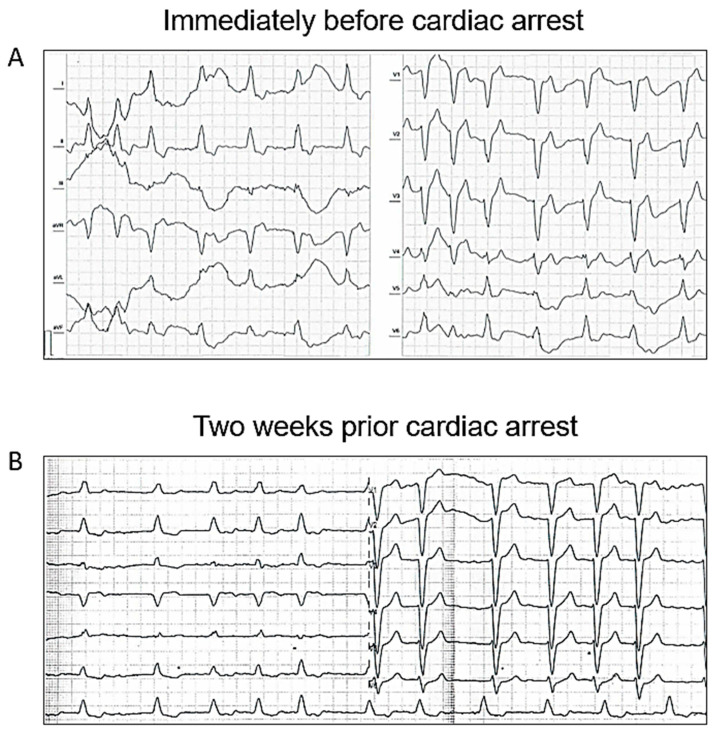
Standard 12-lead electrocardiogram performed immediately before cardiac arrest (**A**). Left bundle branch block and atrial fibrillation were known to be chronic as documented on preoperative 12-lead electrocardiogram obtained 2 weeks prior cardiac arrest (**B**).

**Figure 2:**
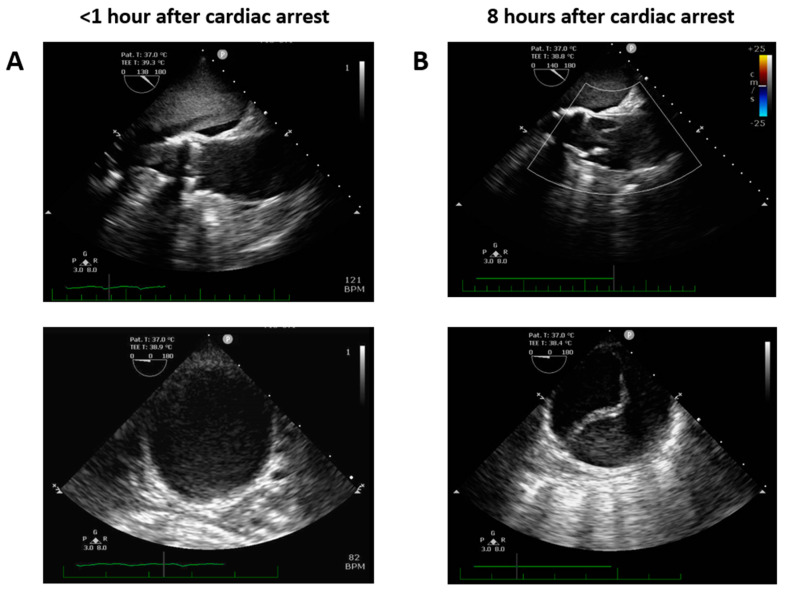
Transesophageal echocardiography focused on ascending and descending thoracic aorta performed early (**A**) and 8 h after cardiac arrest (**B**).

**Figure 3:**
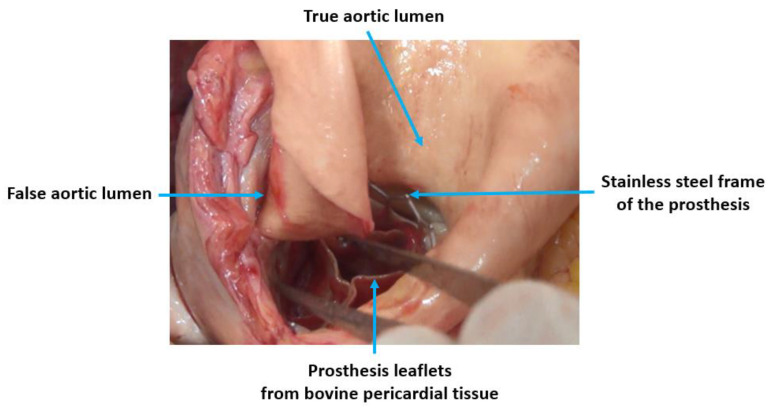
Autopsy finding of thoracic aorta dissection with an entry closely adjacent to the stainless steel frame of the Edwards SAPIEN 3 TAVR prosthesis.

The day of cataract surgery, after premedication (midazolam 3.75 mg and acetazolamide 250 mg orally, and one drop of tropicamide 1% locally), the patient manifested with sudden onset dyspnea accompanied by hypoxemia and elevated systemic arterial pressure (190/120 mmHg). The patient’s status was evaluated as emergent hypertensive crisis with cardiogenic pulmonary edema and was referred to the emergency department. On standard 12-lead electrocardiogram, atrial fibrillation and chronic left bundle branch block were observed (Figure 1). The patient did not react favorably to a standard conservative approach composed of oxygen therapy (delivered by high concentration oxygen mask), intravenously administered loop diuretic (furosemide) and vasodilator (isosorbide dinitrate), and required prompt endotracheal intubation and mechanical ventilation due to ongoing respiratory insufficiency and impaired consciousness (respiratory rate 30 breaths per minute, pulse oximetry peripheral arterial oxygen saturation <50%, Glasgow Coma Scale 8–10 points). Distant inspiratory crackles and an excessive amount of frothy sputum from the endotracheal tube were in agreement with the working diagnosis of cardiogenic pulmonary edema. Immediately following endotracheal intubation, bradycardia and subsequently asystole occurred. Cardiopulmonary resuscitation with advanced life support was initiated using the LUCAS chest compression system (Stryker Medical, Lund, Sweden). Ultrasound performed just before circulatory collapse did not reveal any obvious cause of imminent cardiac arrest, no signs of acute cor pulmonale, pericardial tamponade or any indirect indication of TAD were detected. As cardiac arrest was refractory and the local criteria for extracorporeal cardiopulmonary resuscitation were met, veno-arterial extracorporeal membrane oxygenation (VA-ECMO) was implanted, using a Cardiohelp pump (Getinge, Rastatt, Germany) and standard femoro-femoral cannula configuration (inflow venous 23 Fr, outflow arterial 17 Fr). At the time of circulatory collapse, when the decision to initiate VA-ECMO therapy was made, the arterial level of lactate was 11.8 mmol/L and pH was 6.87. Circulatory collapse to VA-ECMO insertion time was approximately 40 min. Transesophageal echocardiography, used for guidance in VA-ECMO cannula insertion, showed a “standing heart” picture compatible with ongoing pulseless electrical activity, which persisted throughout the entire period of extracorporeal support. Additionally, during approximately the first 8 h on extracorporeal circulatory support, there were still no signs of aortic dissection on transesophageal echocardiography and no intimal flap was detected in the peripheral arteries on regular bedside ultrasound using a vascular probe. Subsequently, the patient became volume-dependent, with decreased systemic vascular resistance, requiring massive fluid resuscitation and high doses of vasopressors to maintain adequate perfusion pressure. Positive crystalloid balance was 4900 mL, positive cumulative fluid balance including colloids and blood derivatives was 10,800 mL. Norepinephrine doses reached 0.7 mcg/kg/min. Regarding initial laboratory blood tests, the patient had normal liver and renal functions, low procalcitonin and C-reactive protein levels, normal total blood cells count with absolute lymphocytosis and relative neutropenia. Plasma level of cardiac troponin I increased from normal value at the time of cardiac arrest to 868,914 ng/L after 12 h, peak value of creatine kinase-MB protein mass was reached 8 h from circulatory collapse (582 mcg/L). After 8 h from cardiac arrest, a clear intimal flap involving the entire length of the thoracic aorta was detected (Figure 2). A conservative therapeutic approach was indicated by the institutional heart team due to poor patient prognosis. VA-ECMO support was withdrawn after 14 h of ongoing pulseless electrical activity. Autopsy revealed Stanford type A TAD causing total obstruction of both coronary arteries, with the entry directly adjacent to the aortic valve prosthesis area (Figure 3). The location of the entry was not typical for spontaneous TAD. No other potential alternative causes of death were identified during autopsy.

## 3. Discussion

The present report describes a case of late Stanford type A TAD related to prior transfemoral TAVR, which presented as refractory in-hospital cardiac arrest with non-shockable rhythm due to coronary arteries obstruction.

Although not entirely clear, upon thorough review of the sequence of clinical signs, along with autopsy findings, we can declare with a high level of certainty that the cause of cardiac arrest was obstruction of the coronary arteries due to TAD. Acute aortic syndrome was likely triggered by increased arterial blood pressure due to stress response preceding ophthalmological intervention, but we cannot entirely exclude that the hypertensive reaction was a secondary response to TAD. The reason that the false aortic lumen was not apparent initially following cardiac arrest may be explained by sufficient VA-ECMO flow via the outflow cannula inserted into the true lumen and the absence of intrinsic left ventricular pulsatility during ongoing electromechanical dissociation. The intimal flap became apparent at the moment when adequate VA-ECMO flow could not be maintained due to relative hypovolemia.

The extreme dynamics of laboratory markers of myocardial necrosis support the hypothesis that the occlusion of, in particular, the left main coronary artery was the lethal mechanism, despite the fact that typical macroscopic signs of myocardial necrosis had not entirely developed on transverse sections of the heart at autopsy due to the short time period. Unresponsiveness to initial therapy of cardiogenic pulmonary edema implies that TAD developed spontaneously and not as a traumatic consequence of cardiopulmonary resuscitation. Nevertheless, chest compression-related aortic injuries should be taken into consideration in cardiac arrest survivors, especially after prolonged mechanical resuscitation and extracorporeal cardiopulmonary resuscitation, which are increasingly used [7]. To the best of our knowledge, cardiopulmonary resuscitation-induced TAD with an entry localized in the area of TAVR prosthesis has not been described in the literature to date. However, Etuk et al. presented a case of cardiopulmonary resuscitation-induced TAD in a patient three months after thoracic endovascular aneurysm repair [8]. Thus, prior endovascular aortic procedure may be a risk factor for cardiopulmonary resuscitation-induced aortic injury.

In 2010, Gerber et al. reported two cases of delayed TAD following TAVR by several days [9]. More recently, rare cases of late type A TAD (5 months and 1 year after TAVR) were documented in octogenarians related to transfemoral implantation of both main types of aortic valves: the balloon-expandable Edwards Sapien Valve (Edwards Lifescience, Irvine, CA, USA) and self-expanding Medtronic CoreValve (Medtronic, Minneapolis, MN, USA) [3,4]. Chest or back pain, typical of acute aortic syndrome, was the leading symptom at the time of initial clinical manifestation in these cases.

In the present case, the patient did not complain of any thoracic pain before circulatory collapse. We may hypothesize that the interval from initial intimal tearing to cardiac arrest due to left main coronary artery obstruction was too short to allow the patient to develop any symptoms or he was not able to provide any meaningful subjective input due to acute respiratory failure. Nevertheless, on 12-lead electrocardiogram obtained immediately before cardiac arrest, in the terrain of the left bundle branch block, we observed some acute ischemic changes suggestive of left main or left anterior descending coronary artery hypoperfusion (Figure 1).

As coronary artery disease is the main cause of cardiac arrest in adults, we considered performing coronary angiography. Taking into account minimal atherosclerotic lesions observed during coronary angiography preceding the TAVR procedure 14 months prior, non-shockable initial rhythm and transport difficulties due to hemodynamic instability, we elected to not proceed with urgent selective coronary angiography. We emphasize that cardiac catheterization would have likely revealed the cause of cardiac arrest. Computed tomography may have also uncovered the diagnosis earlier. Despite the fact that we considered and actively searched for TAD, we did not perform computed tomography angiography as the patient was hemodynamically unstable and the suspicion for TAD was reduced by negative findings on repeated transesophageal echocardiography. Nevertheless, in the present case, an earlier diagnosis would likely not have changed the unfavorable outcome of the patient.

Predisposing factors and triggers of late TAD after TAVR have not extensively been described as cases are scarce. They may differ from the risk factors of TAD after surgical aortic valve replacement, where perioperative manipulation or clamping may also affect the integrity of the ascending aortic wall [10]. We may hypothesize that confounding factors such as uncontrolled hypertension, prosthesis malposition, infective endocarditis and congenital (bicuspid aortic valve) or acquired (corticosteroid therapy) insufficiency of aortic wall connective tissue may increase the risk of TAVR-associated late TAD [11,12]. In the present case, according to medical documentation, arterial hypertension was well compensated, and the diameter of the ascending aorta was only mildly dilated. However, the dimensions of the ascending aorta did not increase over time, preoperative morphology of the aortic valve seemed to be tricuspid and postmortem histological examination did not reveal any apparent insufficiency of aortic wall connective tissue.

## 4. Conclusions

Late Stanford type A TAD with associated sudden cardiac death represents an unusual clinical event in patients who underwent prior TAVR. As the number of TAVR recipients is increasing and the method spreads to younger populations, we should consider this disorder in the differential diagnosis in the context of any sudden unexplained change in clinical status and we should proactively exclude this complication, even in the absence of typical clinical manifestations and indirect echocardiographic signs of acute aortic syndrome. Cardiopulmonary resuscitation-induced aortic injuries should be considered in the differential diagnosis.

## Data Availability

More detailed data regarding present case are available from the corresponding author upon reasonable request.
